# Synthesis and Optoelectronic Properties of Block and Random Copolymers Containing Pendant Carbazole and (Di)phenylanthracene

**DOI:** 10.3390/polym10070721

**Published:** 2018-07-01

**Authors:** Chen-Tsyr Lo, Yohei Abiko, Jun Kosai, Yuichiro Watanabe, Kazuhiro Nakabayashi, Hideharu Mori

**Affiliations:** 1 Department of Organic Materials Science, Graduate School of Organic Materials Science, Yamagata University, 4-3-16, Jonan, Yonezawa 992-8510, Japan; ct.lo@yz.yamagata-u.ac.jp (C.-T.L.); tad67163@st.yamagata-u.ac.jp (J.K.); yuichirow@yz.yamagata-u.ac.jp (Y.W.); nakabayashi.k@yz.yamagata-u.ac.jp (K.N.); 2 Department of Organic Device Engineering, Graduate School of Science and Engineering, Yamagata University, 4-3-16, Jonan, Yonezawa 992-8510, Japan; 99.yohei@gmail.com

**Keywords:** carbazole-containing copolymer, diphenylanthracene, RAFT polymerization, comonomer sequence, fluorescence resonance energy transfer

## Abstract

Synthesis of novel block and random copolymers, containing a carbazole unit and (di)phenylanthracene moiety in the side chains, has been described in this paper. Block and random copolymers composed of 4-bromophenyl vinyl sulfide (BPVS) and *N*-vinylcarbazole (NVC) were initially prepared by reversible addition-fragmentation chain transfer (RAFT) polymerization. Then, anthracene-based groups were introduced on the bromophenyl unit in the carbazole-containing copolymers by Pd-catalyzed coupling to yield functional copolymers with additional (di)phenylanthracene units. The resulting copolymers, having two distinct electronic functionalities, exhibited characteristic fluorescence resonance energy transfer, as confirmed by UV-vis and fluorescence spectra.

## 1. Introduction

Numerous efforts have been devoted to exploring novel polymers having two distinct electronic functionalities, e.g., donor and acceptor chromophores, for a wide range of optoelectronic applications, such as organic light-emitting devices, photovoltaics, and organic field-effect transistors [1,2,3,4,5,6,7,8,9,10]. Most reported polymers are π-conjugated polymers, where an electron donor and an electron acceptor are linked through conjugated bonds in the main chain. Recently, increasing attention has been paid to non-conjugated polymers with a through-space charge transfer effect between a pendant donor and an acceptor, which are suitable for highly efficient blue thermally activated delayed fluorescence [11,12]. The suitability of these polymers with π-conjugated and π-stacked systems for advanced applications is frequently derived from the tunability of these structures according to the location and stacking of the chromophores, electron donating and accepting properties involving HOMO/LUMO energy levels, and interfaces of two distinct functionalities, which give rise to different polymer architectures and sequences (block, random, or alternating copolymers). In addition to band gap engineering and molecular energy-level optimization, the manipulation of energy, electron, and charge transfer between two distinct electronic components is key to achieve further excellent photophysical and optoelectronic properties.

The design and synthesis of anthracene-based polymers are attracting significant attention, due to their characteristic applications as fluorescent labels, photon harvesters, and electro- and photo-luminescent materials. Various polymers with the anthracene unit in the side chain have been introduced by polymerization of anthracene-containing monomers [13] and post-modifications of preformed polymers with reactive sites [14,15]. Anthracene-based polymers and molecules with an additional optoelectronic component are of considerable interest because the combination of two distinct electronic functionalities in a single system provides a great variety of photophysical and optoelectronic properties. In particular, various polymers having anthracene and carbazole units in the main chain [16,17], side chain [18], main/side chains [19], and Langmuir-Blodgett films [20] have been developed by several groups. Attractive features of carbazole-containing polymers include their hole-transporting properties, high charge-carrier properties, and electroluminescent properties [21].

**Scheme 1:**
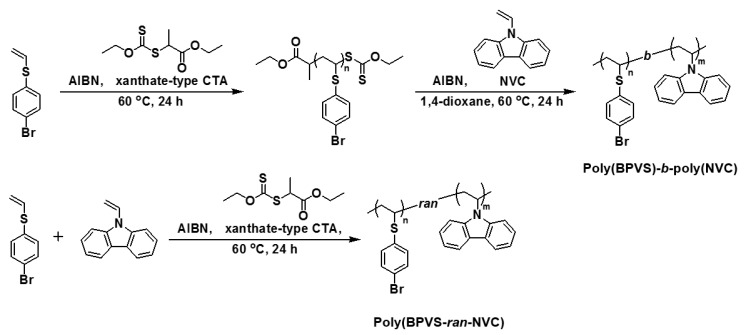
Synthesis of block copolymer and random copolymer by RAFT polymerization using *o*-ethyl-*S*-(1-ethoxycarbonyl)ethyldithiocarbonate as a xanthate-type CTA.

In this paper, we describe the controlled synthesis of block and random copolymers having the (di)phenylanthracene and carbazole group in the side chain. Four copolymers having pendant carbazole and (di)phenylanthracene units in different block and random comonomer sequences were synthesized by reversible addition-fragmentation chain transfer (RAFT) polymerization (Scheme 1). Carbazole and anthracene are both attractive π-stacked units because of their strong excimer-emitting properties [22] and excellent charge-transporting and electrochemical properties [23,24,25,26]. We thus focused on the design and manipulation of energy transfer between carbazole and (di)phenylanthracene units in the block and random copolymers. We previously reported a facile and efficient strategy for the controlled synthesis of block copolymers having carbazole and phenylanthracene units by RAFT polymerization of *N*-vinylcarbazole (NVC) using poly(bromophenyl vinyl sulfide) macro-chain transfer agent, poly(BPVS) macro-CTA, and subsequent palladium-catalyzed coupling [18]. In this study, the same procedure was employed for the synthesis of a block copolymer having two distinct electronic functionalities, in which the carbazole-containing segment is covalently linked with the diphenylanthracene-containing segment. Here, the 9,10-diphenylanthracene unit was selected because it is a well-known blue fluorescent compound with high fluorescence quantum yield (0.97 in THF) [27]. The corresponding random copolymers were also prepared by RAFT copolymerization and subsequent post-modification. This study is aimed at determining the effects of side-chain functionalities having two distinct electronic units, as well as the effect of the comonomer sequence, on energy transfer behavior and characteristic optoelectronic properties. Despite having similar comonomer composition, the copolymers developed in this study exhibited different energy transfer behaviors, evaluated from absorption and fluorescent measurements. In contrast, the block and random copolymers exhibited similar HOMO/LUMO levels and band gaps.

## 2. Experimental Section

### 2.1. Materials

2,2-Azobisisobutyronitrile (AIBN, Kanto Chemical, Tokyo, Japan, 97%) was purified by recrystallization from methanol. 4-Bromophenyl vinyl sulfide (BPVS) was synthesized by the reaction of 1,2-dibromoethane and 4-bromobenzenethiol according to a previously reported method with slight modifications [18,28]. *N*-Vinylcarbazole (NVC, Tokyo Chemical Industry, Tokyo, Japan, >98%) was recrystallized twice from methanol. *O*-Ethyl-*S*-(1-ethoxycarbonyl)ethyldithiocarbonate (CTA) was synthesized by the reaction of potassium ethyl xanthogenate with methyl 2-bromopropionate [29,30]. Other materials were used without further purification.

### 2.2. Synthesis of Block Copolymers

Block copolymers consisting of poly(NVC) and poly(BPVS) having a reactive site were synthesized by RAFT polymerization of NVC using a xanthate-terminated poly(BPVS) macro-CTA, which was prepared by RAFT polymerization of BPVS using CTA (Scheme 1 and Table S1, Supplementary Materials). A detailed description of the synthesis procedure can be found in an earlier publication [18].

Post-modification of poly(BPVS)-*b*-poly(NVC) using 9-anthraceneboronic acid was performed by Pd-catalyzed Suzuki coupling to yield poly(BPVS-An)-*b*-poly(NVC), according to a method reported previously [18]. A THF solution (5 mL) of poly(BPVS)-*b*-poly(NVC) (*M*_n_ = 19400, *M*_w_/*M*_n_ = 1.28, BPVS content = 41%, 100 mg, 0.20 bromo-unit mmol based on the repeating unit), 9-anthraceneboronic acid (0.20 g, 0.91 mmol), and tetrakis(triphenylphosphine) palladium(0) (14.0 mg, 0.012 mmol) was stirred at room temperature under nitrogen. After complete dissolution of starting materials, aqueous Na_2_CO_3_ solution (20 wt % in water, 0.38 mL) was added, and the reaction mixture was refluxed for 48 h in the dark. The reaction mixture was then purified by reprecipitation from a large excess of methanol, and the product was dried under vacuum at room temperature to give poly(BPVS-An)-*b*-poly(NVC) as a yellow solid (>99%). 

The same procedure using 10-phenyl-9-anthraceneboronic acid was employed for the synthesis of poly(BPVS-PAn)-*b*-poly(NVC).

### 2.3. Synthesis of Random Copolymers

AIBN (1.6 mg, 0.01 mmol), CTA (4.4 mg, 0.02 mmol), BPVS (210 mg, 1.0 mmol), NVC (190 mg, 1.0 mmol), and 1,4-dioxane (0.2 mL) were placed in a dry glass ampule equipped with a magnetic stir bar, and then the solution was degassed by three freeze-evacuate-thaw cycles. After the ampule was flame-sealed under vacuum, it was stirred at 60 °C for 24 h. The reaction mixture was purified by reprecipitation from a chloroform solution into a large excess of *n*-hexane and isolated by filtration to give a random copolymer, poly(BPVS-*ran*-NVC) (*M*_n_ = 8200, *M*_w_/*M*_n_ = 1.40, BPVS content = 40%) as a white solid (Scheme 1 and Table S2, Supplementary Materials). Post-modification of the random copolymer, poly(BPVS-*ran*-NVC), was conducted by Suzuki coupling using the above-mentioned method. The Pd-catalyzed reaction of 9-anthraceneboronic acid with the bromophenyl moieties of poly(BPVS-*ran*-NVC) yielded poly(BPVS-An-*ran*-NVC) (>99%). Similarly, the Pd-catalyzed coupling of 10-phenyl-9-anthraceneboronic acid with the bromophenyl moieties in poly(BPVS-*ran*-NVC) yielded poly(BPVS-PAn-*ran*-NVC) as a yellow solid (>99%).

^1^H NMR spectra of these block and random copolymers and the solubility each component are shown in Figures S1–S3 and Table S3, respectively (see Supplementary Materials).

### 2.4. Instrumentation

^1^H (400 MHz) and ^13^C NMR (100 MHz) spectra were recorded on a JEOL JNM-ECX400 instrument (JEOL, Tokyo, Japan). Elemental analysis was performed on a Perkin-Elmer 2400 II CHNS/O analyzer (Perkin-Elmer, Waltham, MA, USA). The number-averaged molecular weight (*M*_n_) and molecular weight distribution (*M*_w_/*M*_n_) were estimated by size-exclusion chromatography (SEC) using a Tosoh HPLC HLC-8220 (Tosoh, Tokyo, Japan) system equipped with refractive index and ultraviolet detectors at 40 °C. The column set-up was as follows: four consecutive hydrophilic vinyl polymer-based gel columns [TSK-GELs (bead size, exclusion limited molecular weight): α-M (13 μm, > 1 × 10^7^), α-4000 (10 μm, 4 × 10^5^), α-3000 (7 μm, 9 × 10^4^), α-2500 (7 μm, 5 × 10^3^), 30 cm each] and a guard column [TSK-guard column α, 4.0 cm]. The system was operated at a flow rate of 1.0 mL/min using DMF containing 10 mM LiBr as the eluent. Polystyrene standards (Tosoh, Tokyo, Japan) ranging from 1050 to 1,090,000 were employed for calibration. UV-vis spectra were recorded using a JASCO V-630BIO UV-vis spectrophotometer (JASCO, Tokyo, Japan). Fluorescence spectra were obtained from a JASCO FP-6100 spectrofluorophotometer (JASCO, Tokyo, Japan). The molecular energy level was estimated by density functional theory (DFT) at the RB3LYP/6–311G+(d,p) (Gaussian Inc., Wallingford, CT, USA) [31]. 

Cyclic voltammetry (CV) experiments were performed on a BAS electrochemical analyzer (model 660C, BAS, Tokyo, Japan) in anhydrous acetonitrile solutions with 0.1 M tetrabutylammonium hexafluorophosphate as a supporting electrolyte. A three-electrode cell with platinum electrodes as both the counter and working electrodes was used. Ag/Ag^+^ (Ag in 0.01 M AgNO_3_ solution) was used as the reference electrode. Ferrocene/Ferrocenium (Fc/Fc^+^) was used as the internal standard. Tapping-mode scanning force microscopy (SFM) analysis was performed with an Agilent AFM 5500 (Agilent Technologies, Santa Clara, CA, USA), using micro-fabricated cantilevers with a force constant of ~34 N/m. The sample was prepared as follows: a droplet of the THF solution of the copolymer was cast onto a mica substrate and dried at room temperature.

## 3. Results and Discussion

### 3.1. Synthesis of Block and Random Copolymers

**Scheme 2:**
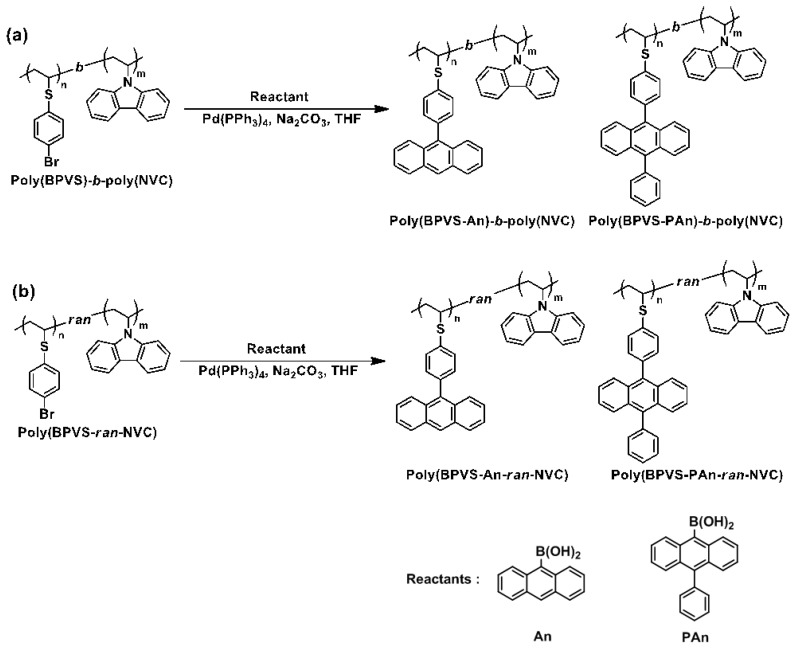
Synthesis of (**a**) block copolymers and (**b**) random copolymers with (di)phenylanthracene moieties.

Block and random copolymers containing the carbazole unit and (di)phenylanthracene moiety in their side chains were synthesized by RAFT polymerization (Scheme 1 and Scheme 2) to develop an optimal sequence (block or random sequence) manipulating efficient energy transfer. In general, there are two ways to introduce a functionality to a polymer backbone: polymerization of a prefunctionalized monomer and attachment of the desired functionality to a prepolymer with a reactive site via postmodification. In this study, RAFT polymerization of NVC was employed to produce a carbazole-containing segment, whereas the attachment of an anthracene-based unit to preformed poly(BPVS) with a reactive active site was achieved by Pd-catalyzed coupling. This approach was selected to afford the following three advantages: (a) applicability of bulky anthracene and phenylanthracene for less steric hindrances in the synthesis of block and random copolymers; (b) ability to create copolymers with random comonomer distributions due to less bulkiness of the BPVS monomer compared to anthracene-containing monomers; and (c) ability to use the same copolymers for the attachment of different functionalities. These advantages may help us understand the effects of side-chain functionalities having two distinct electronic units as well as of the comonomer sequence on the energy transfer behavior and characteristic optoelectronic properties.

A block copolymer consisting of poly(NVC) as an electron acceptor segment and poly(BPVS) was synthesized by RAFT polymerization. The BPVS/NVC ratio in the feed, the molecular weight of the poly(BPVS) macro-CTA, and the monomer-to-CTA ratio were adjusted to obtain copolymers having similar comonomer compositions and molecular weights. The xanthate-terminated poly(BPVS) with relatively low molecular weight, which was prepared by RAFT polymerization of BPVS using a xanthate-type CTA, was employed as a macro-CTA. NVC polymerization was conducted in dry 1,4-dioxane using the xanthate-terminated poly(BPVS) as a macro-CTA at [NVC]_0_/[macro-CTA]_0_ = 100 at 60 °C for 24 h (Scheme 1 and Table S1, Supplementary Materials). The resulting poly(BPVS)-*b*-poly(NVC), with reasonable molecular weight, polydispersity, and comonomer composition (*M*_n_ = 19400, *M*_w_/*M*_n_ = 1.28, BPVS content = 41%) was used for the postmodifications. Two boronic acid derivatives, 10-phenyl-9-phenylanthraceneboronic acid (PAn) and 9-anthraceneboronic acid (An), were employed to afford block copolymers with the diphenylanthracene unit and phenylanthracene moiety, respectively (Scheme 2). Incorporation of the anthracene unit into the poly(BPVS) segment in the block copolymer was carried out with a large excess of boronic acid derivatives in the presence of Pd catalyst and Na_2_CO_3_ in THF/water to yield poly(BPVS-PAn)-*b*-poly(NVC) and poly(BPVS-An)-*b*-poly(NVC), respectively (Table 1).

A random copolymer was also synthesized via the RAFT process, followed by Pd-catalyzed coupling. RAFT copolymerization of NVC and BPVS was conducted using the xanthate-type CTA in dry 1,4-dioxane using at [BPVS]_0_/[NVC]_0_ = 50/150 and [CTA]_0_/[AIBN]_0_ = 2/1 at 60 °C for 24 h (Scheme 1 and Table S2, Supplementary Materials) to obtain a random copolymer with pre-determined comonomer composition (BPVS content = 40%) and relatively low polydispersity (*M*_w_/*M*_n_ = 1.40). Postmodification of the random copolymer using the anthracene-based boronic acids (PAn and An) yielded poly(BPVS-PAn-*ran*-NVC) and poly(BPVS-An-*ran*-NVC), respectively. All the copolymers showed reasonable molecular weights and good solubility in common organic solvents, such as THF and chloroform, ensuring reasonable film formation through the solution process, such as spin-coating and drop-casting. Therefore, based on the side-chain engineering strategy, the resulting copolymers successfully inherited the characteristics of the carbazole and (di)phenylanthracene units. Unlike the block copolymers, where the pendant (di)phenylanthracene unit is separated from the carbazole unit, the (di)phenylanthracene unit is close to the carbazole unit in the random copolymers. Hence, the influence of distance between the pendant carbazole and (di)phenylanthracene units on the energy transfer can be explored.

### 3.2. Optoelectronic Properties of Block and Random Copolymers

**Figure 1:**
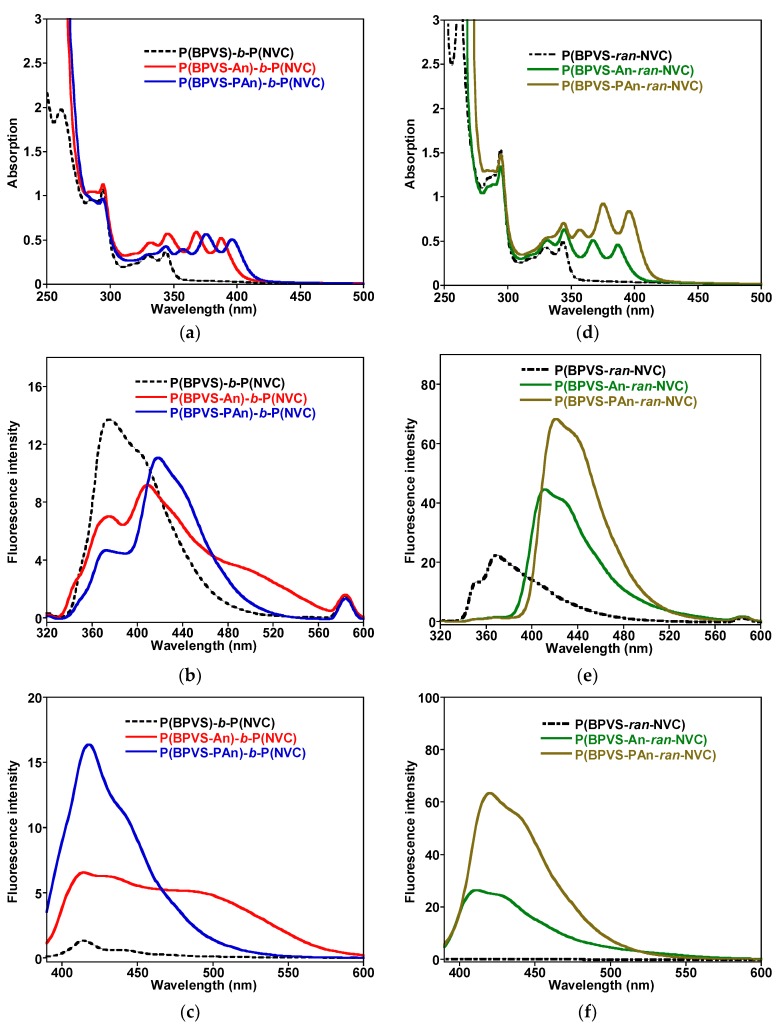
UV-vis absorption spectra (conc. = 1.0 × 10^−4^ carbazole unit mol/L in THF) of (**a**) block copolymers and (**d**) random copolymers, and fluorescence spectra (conc. 1.0 × 10^−5^ carbazole unit mol/L in THF) of (**b**,**c**) block copolymers (excitation wavelength: (**b**) 294 and (**c**) 369 nm, respectively) and (**e**,**f**) random copolymers (excitation wavelength: (**e**) 294 and (**f**) 369 nm, respectively).

Both the absorption and fluorescence properties of the two block copolymers with the carbazole and (di)phenylanthracene units in the side chain and the two random copolymers were investigated in THF (Figure 1). Figure 1a shows that poly(BPVS-PAn)-*b*-poly(NVC) exhibits the first absorption band at around 260 nm, assigned to the anthracene moiety, and a second absorption in the range of 350–430 nm. The latter adsorption may stem from the adjacent anthracene moieties, which is shifted toward longer wavelengths when compared to that of poly(BPVS-An)-*b*-poly(NVC). The peaks attributed to the poly(NVC) segment are detected at 300–350 nm in both the block copolymers and the pristine block copolymer, poly(BPVS)-*b*-poly(NVC). A similar tendency was observed in the spectra of random copolymers (Figure 1d). Here, for both block and random copolymers, it is found that the absorption of the carbazole unit at 300–350 nm has no significant interaction with that of anthracene unit at 330–430 nm, indicating the limited overlapping structure.

Fluorescence spectra of the (di)phenylanthracene-containing block copolymers excited at 294 nm, corresponding to the absorption wavelength of the carbazole unit, are shown in Figure 1b. The emission of poly(BPVS-PAn)-*b*-poly(NVC) is observed at 370 and 440 nm, attributed to the carbazole unit and the anthracene moiety, respectively. In addition to these peaks, a broad tailing of the peak is detected up to ~550 nm. Similarly, poly(BPVS-An)-*b*-poly(NVC) exhibits fluorescence peaks at both 370 and 430 nm with a broad tailing of the peak up to ~600 nm. For the random copolymers (Figure 1e, excitation wavelength: 294 nm), strong fluorescence signals corresponding to the anthracene groups are observed in a range of 380–520 nm, whereas the intensity of the fluorescence peaks attributed to the carbazole unit at less than 380 nm is too weak to be visible. Compared to the spectra of the block copolymers in Figure 1b, the fluorescence intensity of the random copolymers was almost 5-times higher than that of the block copolymers. These behaviors could be explained by fluorescence resonance energy transfer (FRET), which is referred to as an energy transfer between a FRET donor and acceptor. In general, the FRET efficiency is sensitively and inversely proportional to the distance between fluorescent donors and acceptors. For random copolymers, the anthracene-based acceptor unit and carbazole-based donor unit have less limitation from the main chain, and thus greater ability to interact with each other, leading to efficient energy transfer from the carbazole to the (di)phenylanthracene groups. The DFT calculation of carbazole and (di)phenylanthracene moieties supported efficient FRET from the carbazole to the (di)phenylanthracene moieties (Figure S4). The (di)phenylanthracene moieties exhibited much deeper LUMO levels (−2.09 and −2.08 eV) and smaller energy band gaps (3.49 and 3.43 eV) than those of the carbazole moiety (−1.10 and 4.53 eV, respectively). Judging from the DFT results, FRET from the carbazole to the (di)phenylanthracene moieties was a consistent phenomenon. In contrast, because of the confinement of the backbone, the distance between the anthracene-based segment and carbazole-based donor in the block copolymers is not short enough, resulting in insufficient energy transfer between the donor-acceptor moieties. High fluorescence intensity can be attributed to the well-defined donor-acceptor domains with limited overlapping structures. In the organized nanostructures of carbazole and (di)phenylanthracene in the prepared copolymers, especially in the random copolymer systems, numerous photo-generated excitons did not easily recombine, resulting in significant emission peaks in the systems.

For excitation at 369 nm, corresponding to the absorption wavelength of the anthracene unit, no significant energy transfer was observed from the anthracene unit to the carbazole unit in both block and random copolymers, resulting in a drastic decline in the intensities of the anthracene-based fluorescent peaks (Figure 1c,f). Besides, the incorporation of phenylanthracene (PAn) units in both block and random copolymers, instead of the anthracene (An) units, resulted in significantly red-shifted adsorption peaks (Figure 1a,d) and significant emission throughout the fluorescence spectra (Figure 1b,c,e,f). This implies that the diphenylanthracene side chains formed by the incorporation of PAn units with intrinsic higher chain mobility, compared to the more rigid phenylanthracene pendant groups incorporated by An units, may have greater interaction with the carbazole units, leading to more significant energy transfer in the system.

**Figure 2:**
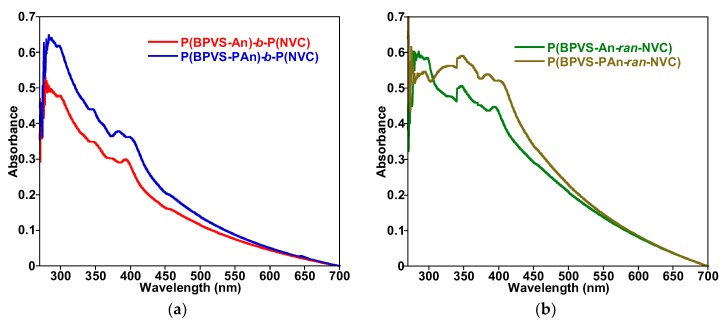
UV-vis absorption spectra of (**a**) block copolymers and (**b**) random copolymers in solid thin films. The sample was prepared by spin-coating from THF solution (10 mg/mL).

In order to find an optimal sequence for manipulating efficient energy transfer, the solid-state absorption of the block and random copolymers was also evaluated in the thin film states. Figure 2 shows that the absorption spectra in the thin film states are apparently red-shifted compared to those in the THF solution state, suggesting a specific conformation after conversion into the thin film state. It is also observed that the incorporation of PAn units in both block and random copolymers, instead of An units, resulted in more red-shifted adsorption peaks of adjacent anthracene moieties, compared to those of An units. Higher intrinsic mobility of the PAn units afforded greater interaction between the donor and acceptor moieties even in the spin-coating process, resulting in significantly red-shifted signals. Moreover, broad tailing of the absorption peak up to ~700 nm was detected, regardless of the comonomer sequence or the substituting group of the anthracene unit. Furthermore, no significant difference was detected in the solid-state conformation between the block and random copolymers, which is apparently distinct from the solution state.

**Figure 3:**
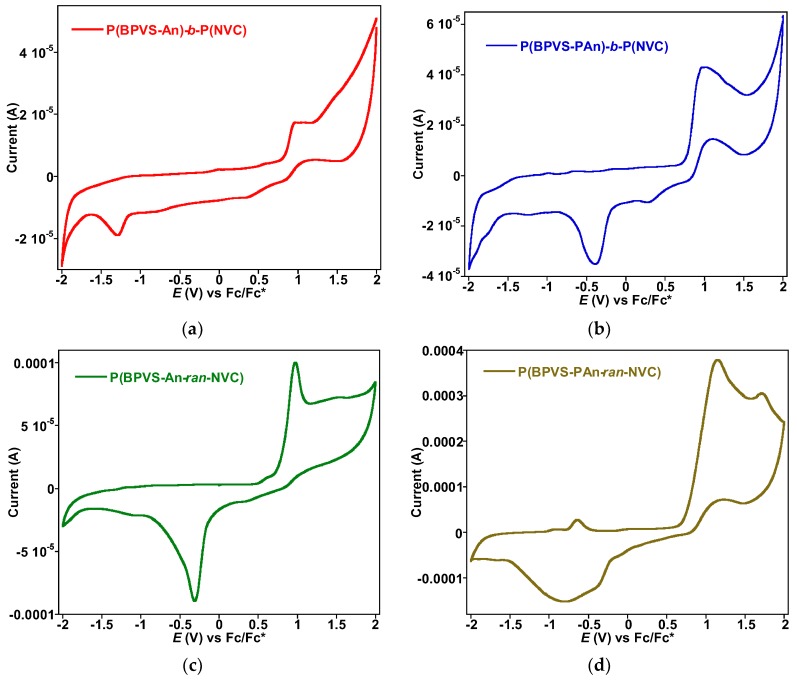
CV spectra of (**a**) poly(BPVS-An)-*b*-poly(NVC); (**b**) poly(BPVS-PAn)-*b*-poly(NVC); (**c**) poly(BPVS-An-*ran*-NVC); and (**d**) poly(BPVS-PAn-*ran*-NVC).

To evaluate the HOMO/LUMO levels and the band gaps of the studied block and random copolymers, cyclic voltammetry (CV) measurements were performed in anhydrous acetonitrile solution containing 0.1 M tetrabutylammonium hexafluorophosphate. The CV profiles of the block and random copolymers (Figure 3) show that the HOMO levels of each material were evaluated from the onset oxidative potentials (*E*_ox_^onset^). Table 2 shows that the HOMO levels of the studied block and random copolymers were estimated to lie between −5.51 and −5.57 eV, which can be mainly revived from the intrinsic electron-donating tendency of the carbazole-based unit. The LUMO levels ranging from −2.84 to −2.93 eV, which were determined from the HOMO levels and the optical band gap (*E*_g_^opt^), reflect the electron-accepting nature of the anthracene-based units. Both the block and random copolymers maintained the intrinsic energy levels of employing moieties, which is consistent with the analysis of the absorption spectra, where no significant new peak was performed in either system. In our system, the pendant carbazole donor and (di)phenylanthracene acceptor are physically separated, leading to a small overlap of the HOMO and LUMO levels. The spatial π–π interaction between the physically separated donor and acceptor units in the random copolymer should be distinct from that in the block copolymer. Nevertheless, no significant difference was detected in the HOMO/LUMO levels and in the band gaps between the block copolymers and the random copolymers, suggesting that the comonomer sequence has limited influence on the electrochemical properties.

### 3.3. Assembled Structures of Block and Random Copolymers

**Figure 4:**
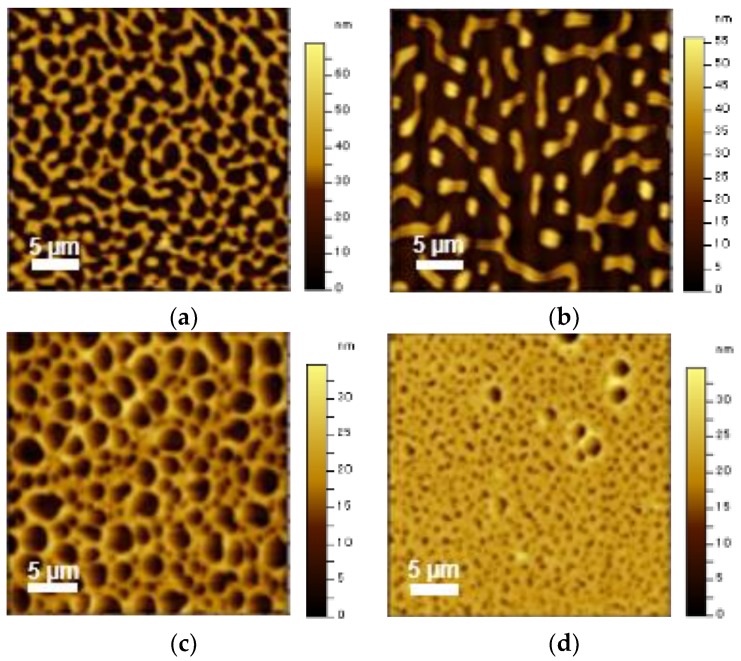
SFM height images of (**a**) poly(BPVS-An)-*b*-poly(NVC); (**b**) poly(BPVS-PAn)-*b*-poly(NVC); (**c**) poly(BPVS-An-*ran*-NVC); and (**d**) poly(BPVS-PAn-*ran*-NVC). The films were prepared by drop-casting onto mica substrates (conc. = 2.0 mg/mL in THF).

SFM measurement of the drop-cast films was conducted to obtain an in-depth understanding of the nanostructure in the studied block and random copolymers (Figure 4). The samples were prepared by drop-casting onto freshly cleaved mica from each THF solutions (2.0 mg/mL). Based on the solid-state adsorption spectra in Figure 2, it is assumed that the conformation is independent of the comonomer sequence. However, though the molecular conformation is similar, slight differences in the dewetting behavior and thin-film morphology were observed between the block and random copolymers.

Compared to the images of the block copolymers with severe microphase separation, the images of the random copolymers prepared by the same drop-casting method showed more well-defined films. This phenomenon may be due to the more effective energy transfer in the random copolymer system, which was also observed in the fluorescence spectra (Figure 1e,f). The most well-defined films were achieved in the random copolymer employing PAn segments, and efficient intermolecular energy transfer was confirmed to be due to the fine-tuned thin-film morphology (Figure 4d).

## 4. Conclusions

The control of the block and random sequences of copolymers is an essential factor in the manipulation of the energy transfer, because they affect the distance between the fluorescent donor and acceptor. Two block copolymers, poly(BPVS-PAn)-*b*-poly(NVC) and poly(BPVS-An)-*b*-poly(NVC), and corresponding random copolymers, were prepared by RAFT polymerization followed by Pd-catalyzed coupling. The UV absorption and fluorescent spectra for the block and random copolymers having the carbazole unit and (di)phenylanthracene moiety in the side chains indicated that characteristic fluorescence resonance energy transfer took place, depending on the combination of the two distinct electronic functionalities and the comonomer sequence. The CV results suggested the limited influence of comonomer sequence on the intrinsic energy levels. SFM measurements also indicated that the thin-film morphology was affected by the intermolecular energy transfer. In conclusion, the random copolymer poly(BPVS-PAn-*ran*-NVC) presented the most effective energy transfer properties, resulting in the greatest fluorescence emission and best film morphology.

## Figures and Tables

**Table 1:** Characterization data of block and random copolymers prepared by RAFT polymerization, followed by postmodification ^a^.

Copolymer	Before Postmodification			After Postmodification		
	*M*_n_^b^	*M*_w_/*M*_n_^b^	Composition ^c^ (BPVS/NVC)	Reactant ^d^	Yield ^e^	*M*_n_^f^ (Calcd)
poly(BPVS-An)-*b*-poly(NVC)	19,400	1.28	41/59	An	>99	23,000
poly(BPVS-PAn)-*b*-poly(NVC)				PAn	>99	25,800
poly(BPVS-An-*ran*-NVC)	8200	1.40	40/60	An	>99	9700
poly(BPVS-PAn-*ran*-NVC)				PAn	>99	10,800

^a^ See Scheme 1 and Scheme 2 and Tables S1–S2 (Supplementary Materials) for detailed polymerization conditions; ^b^ Number-average molecular weight (*M*_n_) and molecular weight distribution (*M*_w_/*M*_n_) were measured by size-exclusion chromatography (SEC) using polystyrene standards in DMF (10 mM LiBr); ^c^ Calculated by elemental analysis; ^d^ An = 9-anthraceneboronic acid, Pan = 10-phenyl-9-anthraceneboronic acid. The reaction was conducted in the presence of Na_2_CO_3_ (10 equiv for the bromo unit) and Pd(PPh_3_)_4_ (6 mol % for the bromo unit) in THF at 100 °C for 48 h; ^e^ Methanol-insoluble part; ^f^ Calculated from *M*_n_ and BPVS content of the precursors and yield of the postmodification.

**Table 2:** Electrochemical properties of polymers.

Sample	HOMO ^a,b^ (eV)	LUMO ^a,c^ (eV)	*E*_g_^el d^ (eV)
poly(BPVS-An)-*b*-poly(NVC)	−5.57	−2.86	2.71
poly(BPVS-PAn)-*b*-poly(NVC)	−5.52	−2.84	2.68
poly(BPVS-An-*ran*-NVC)	−5.54	−2.93	2.61
poly(BPVS-PAn-*ran*-NVC)	−5.51	−2.88	2.63

^a^ vs. Fc/Fc^+^; ^b^ HOMO = −(*E*_ox_^onset^ + 4.8) (eV); ^c^ LUMO = HOMO + *E*_g_^opt^ (eV); ^d^ *E*_g_^el^ = LUMO − HOMO (eV).

## Supplementary Materials

The following are available online at http://www.mdpi.com/2073-4360/10/7/721/s1, Figure S1: ^1^H NMR spectrum of poly(BPVS)-*b*-poly(NVC) in CDCl_3_, Figure S2: ^1^H NMR spectrum of poly(BPVS-*ran*-NVC) in CDCl_3_, Figure S3: ^1^H NMR spectra of (a) poly(BPVS-An-*ran*-NVC) and (b) poly(BPVS-PAn-*ran*-NVC) in CDCl_3_, Figure S4: Spatial distributions of the HOMOs and LUMOs of carbazole and (di)phenylanthracene moieties, Table S1: Synthesis of poly(BPVS)-*b*-poly(NVC) at 60 °C for 24 h in bulk, Table S2: RAFT copolymerization of BPVS with NVC using xanthate-type CTA, Table S3: Solubility of poly(BPVS-An), poly(BPVS-PAn), and poly(NVC).

## References

[1] Segalman R.A., McCulloch B., Kirmayer S., Urban J.J. (2009). Block copolymers for organic optoelectronics. Macromolecules.

[2] Laquai F., Park Y.-S., Kim J.-J., Basche T. (2009). Excitation energy transfer in organic materials: From fundamentals to optoelectronic devices *Macromol*. Rapid Commun..

[3] Inganas O., Zhang F., Andersson M.R. (2009). Alternating polyfluorenes collect solar light in polymer photovoltaics. Acc. Chem. Res..

[4] Sonar P., Singh S.P., Leclere P., Surin M., Lazzaroni R., Lin T.T., Dodabalapur A., Sellinger A. (2009). Synthesis, characterization and comparative study of thiophene-benzothiadiazole based donor-acceptor-donor (D-A-D) materials. J. Mater. Chem..

[5] Guenes S., Neugebauer H., Sariciftci N.S. (2007). Conjugated polymer-based organic solar cells. Chem. Rev..

[6] Zhang X., Li Z.-C., Li K.-B., Lin S., Du F.-S., Li F.-M. (2006). Donor/acceptor vinyl monomers and their polymers: Synthesis, photochemical and photophysical behavior. Prog. Polym. Sci..

[7] Cheng Y.-J., Yang S.-H., Hsu C.-S. (2009). Synthesis of conjugated polymers for organic solar cell applications. Chem. Rev..

[8] Ye L., Hu H., Ghasemi M., Wang T., Collins B.A., Kim J.-H., Jiang K., Carpenter J.H., Li H., Li Z. (2018). Quantitative relations between interaction parameter, miscibility and function in organic solar cells. Nat. Mater..

[9] Ye L., Jiao X., Zhang H., Li S., Yao H., Ade H., Hou J. (2015). 2D-Conjugated Benzodithiophene-Based Polymer Acceptor: Design, Synthesis, Nanomorphology, and Photovoltaic Performance. Macromolecules.

[10] Ye L., Zhang S., Zhao W., Yao H., Hou J. (2014). Highly Efficient 2D-Conjugated Benzodithiophene-Based Photovoltaic Polymer with Linear Alkylthio Side Chain. Chem. Mater..

[11] Shao S., Hu J., Wang X., Wang L., Jing X., Wang F. (2017). Blue thermally activated delayed fluorescence polymers with nonconjugated backbone and through-space charge transfer effect. J. Am. Chem. Soc..

[12] Zeng X., Luo J., Zhou T., Chen T., Zhou X., Wu K., Zou Y., Xie G., Gong S., Yang C. (2018). Using ring-opening metathesis polymerization of norbornene to construct thermally activated delayed fluorescence polymers: High-efficiency blue polymer light-emitting diodes. Macromolecules.

[13] Demetriou M., Krasia-Christoforou T. (2012). Well-defined diblock copolymers possessing fluorescent and metal chelating functionalities as novel macromolecular sensors for amines and metal ions. J. Polym. Sci. Part A Polym. Chem..

[14] Shenhar R., Sanyal A., Uzun O., Rotello V.M. (2004). Anthracene-functionalized polystyrene random copolymers: Effects of side-chain modification on polymer structure and behavior. Macromolecules.

[15] Howe D.H., McDaniel R.M., Magenau A.J.D. (2017). From click chemistry to cross-coupling: Designer polymers from one efficient reaction. Macromolecules.

[16] Du J., Fang Q., Bu D., Ren S., Cao A., Chen X. (2005). A new poly(fluorene-*co*-carbazole) with a large substituent group at the 9-position of the carbazole moiety: An efficient blue emitter. Macromol. Rapid Commun..

[17] Chen Q., Han B.-H. (2009). Prepolymerization and postpolymerization functionalization approaches to fluorescent conjugated carbazole-based glycopolymers via click chemistry. J. Polym. Sci. Part A Polym. Chem..

[18] Nakabayashi K., Abiko Y., Mori H. (2013). Raft polymerization of s-vinyl sulfide derivatives and synthesis of block copolymers having two distinct optoelectronic functionalities. Macromolecules.

[19] Mori H., Tando I., Tanaka H. (2010). Synthesis and optoelectronic properties of alternating copolymers containing anthracene unit in the main chain by radical ring-opening polymerization. Macromolecules.

[20] Ray K., Misra T.N. (1999). Energy transfer between carbazole and anthracene moieties organised in langmuir–blodgett films *J*. Phys. Chem. Solids.

[21] Grazulevicius J.V., Strohriegl P., Pielichowski J., Pielichowski K. (2003). Carbazole-containing polymers: Synthesis, properties and applications. Prog. Polym. Sci..

[22] Hoffmann S.T., Schrögel P., Rothmann M., Albuquerque R.Q., Strohriegl P., Köhler A. (2011). Triplet excimer emission in a series of 4,4′-bis(*N*-carbazolyl)-2,2′-biphenyl derivatives. J. Phys. Chem. B.

[23] Chen X., Liao J.-L., Liang Y., Ahmed M.O., Tseng H.-E., Chen S.-A. (2002). High-efficiency red-light emission from polyfluorenes grafted with cyclometalated iridium complexes and charge transport moiety. J. Am. Chem. Soc..

[24] Thomas K.R.J., Lin J.T., Tao Y.-T., Ko C.-W. (2001). Light-emitting carbazole derivatives:  Potential electroluminescent materials. J. Am. Chem. Soc..

[25] Yang W.J., Kim D.Y., Jeong M.-Y., Kim H.M., Lee Y.K., Fang X., Jeon S.-J., Cho B.R. (2005). Two-photon absorption properties of 2,6-bis(styryl)anthracene derivatives: Effects of donor–acceptor substituents and the π center. Chem. Eur. J..

[26] Kim Y.H., Jeong H.C., Kim S.H., Yang K., Kwon S.K. (2005). High-purity-blue and high-efficiency electroluminescent devices based on anthracene. Adv. Funct. Mater..

[27] Iida A., Yamaguchi S. (2009). Intense solid-state blue emission with a small stokes’ shift: Pi-stacking protection of the diphenylanthracene skeleton. Chem. Commun..

[28] Abiko Y., Matsumura A., Nakabayashi K., Mori H. (2014). Thermoresponsive core–shell nanoparticles with cross-linked π-conjugate core based on amphiphilic block copolymers by raft polymerization and palladium-catalyzed coupling reactions. Polymer.

[29] Destarac M., Brochon C., Catala J.M., Wilczewska A., Zard S.Z. (2002). Macromolecular design via the interchange of xanthates (madix): Polymerization of styrene with *o*-ethyl xanthates as controlling agents. Macromol. Chem. Phys..

[30] Maki Y., Mori H., Endo T. (2007). Controlled raft polymerization of *n*-vinylphthalimide and its hydrazinolysis to poly(vinyl amine). Macromol. Chem. Phys..

[31] Frisch M.J., Trucks G.W., Schlegel H.B., Scuseria G.E., Robb M.A., Cheeseman J.R., Scalmani G., Barone V., Mennucci B., Petersson G.A. (2013). Gaussian 09, Revision D.01.

