# Supplementary material for: Synthesis and Optoelectronic Properties of Block and Random Copolymers Containing Pendant Carbazole and (Di)phenylanthracene
Source: Polymers (Basel). 2018 Jul 1;10(7):721. doi: 10.3390/polym10070721 (PMC6403895; doi:10.3390/polym10070721)
Supplement: Supplementary file 1 [file polymers-10-00721-s001.pdf]

Supporting Information

Synthesis and Optoelectronic Properties of Block and Random Copolymers Having Pendant Carbazole and (Di)phenylanthracene

Chen-Tsyr Lo†, *Yohei Abiko*‡, *Jun Kosai*†, *Yuichiro Watanabe*†, *Kazuhiro Nakabayashi*†‡, *Hideharu Mori**†‡

†Department of Organic Materials Science, Graduate School of Organic Materials Science

‡Department of Organic Device Engineering, Graduate School of Science and Engineering Yamagata University, 4-3-16, Jonan, Yonezawa, 992-8510, Japan

*To whom correspondence should be addressed. e-mail: h.mori@yz.yamagata-u.ac.jp Phone:+81-238-26-3765, Fax: +81-238-26-3092

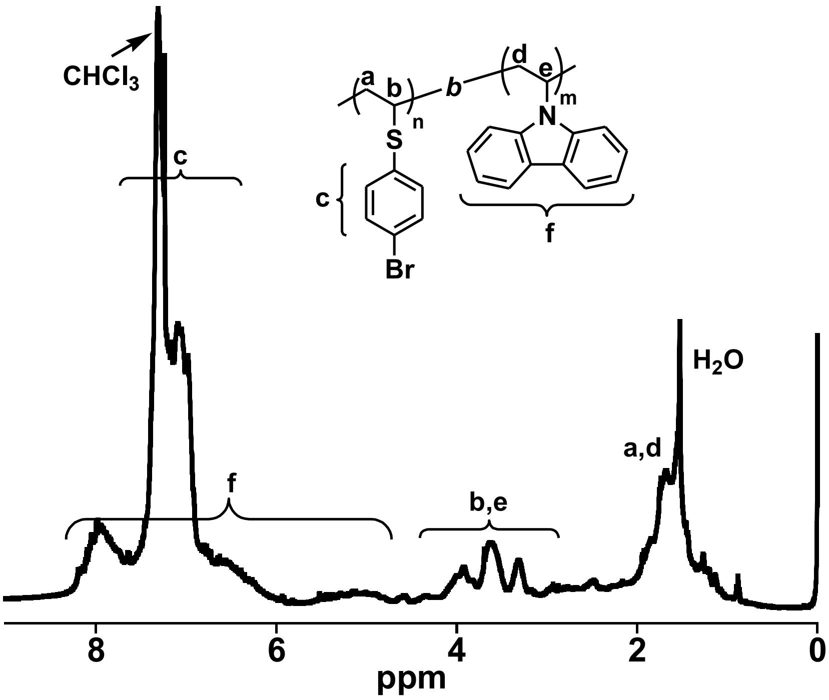

Figure S1. ^{1}H NMR spectrum of poly(BPVS)-*b*-poly(NVC) in $CDCl_3$.

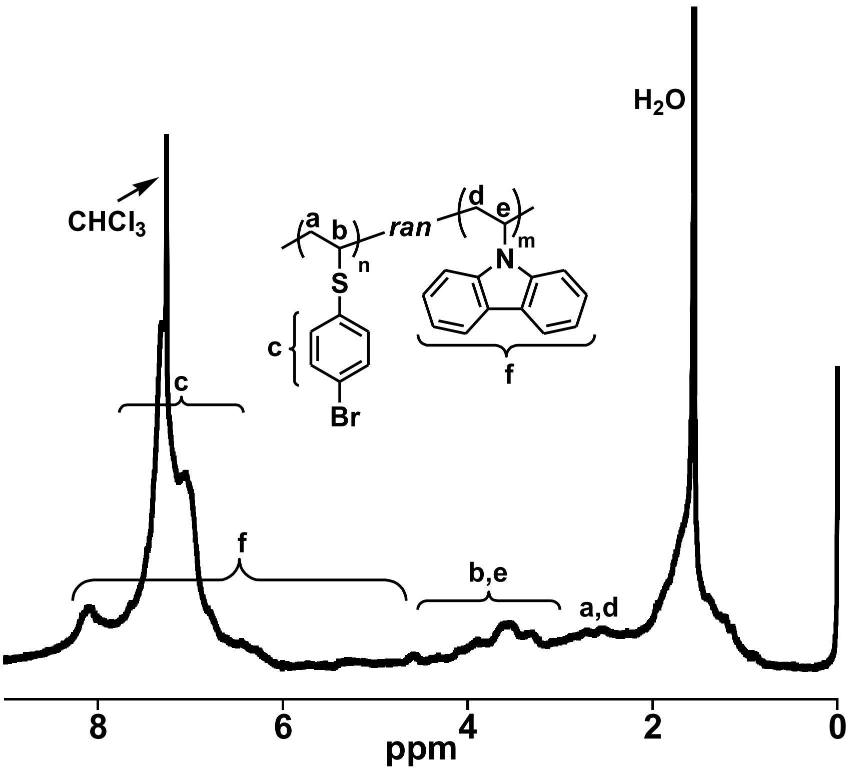

Figure S2. ^{1}H NMR spectrum of poly(BPVS-*ran*-NVC) in $CDCl_3$.

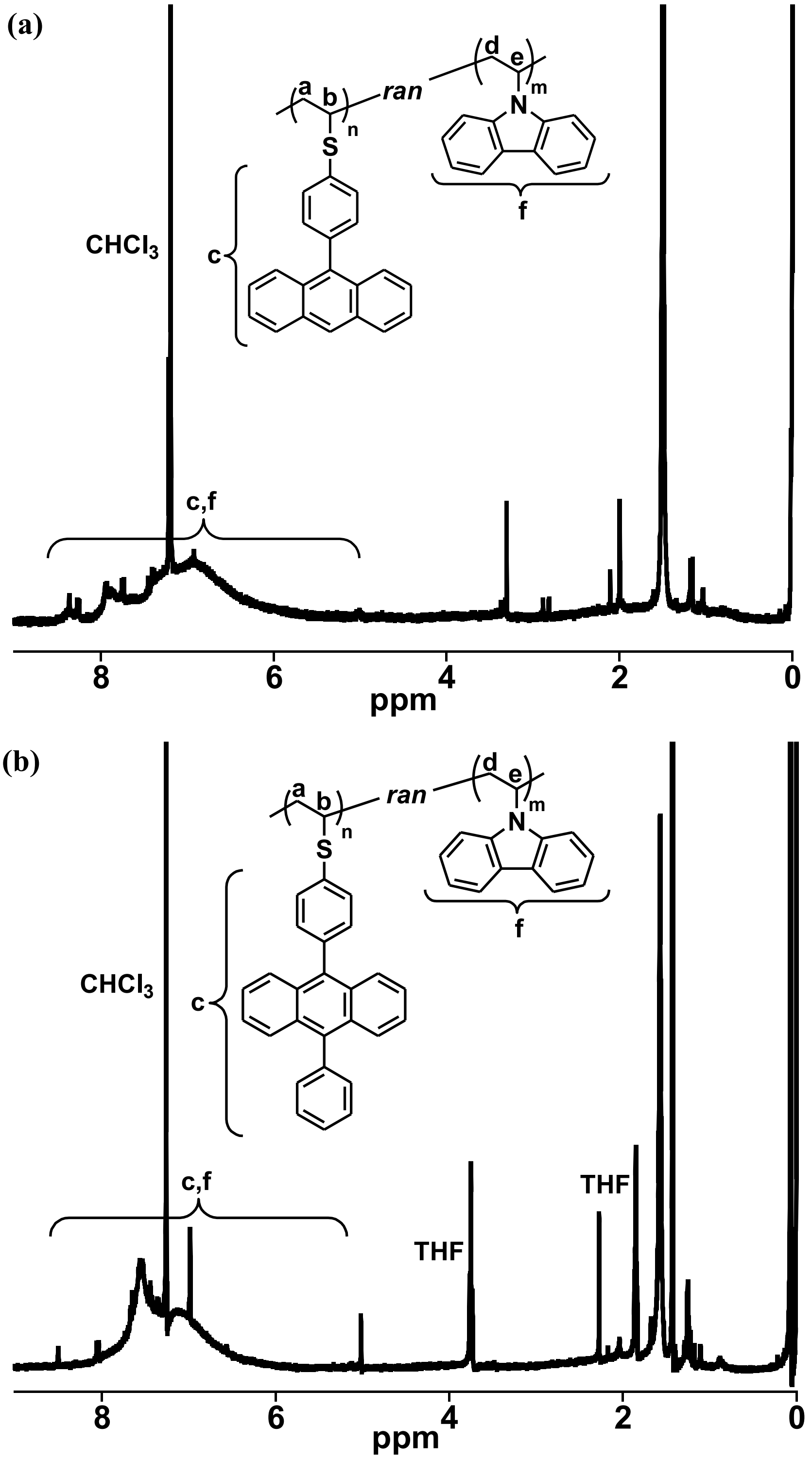

Figure S3. ^{1}H NMR spectra of (a) poly(BPVS-An-*ran*-NVC) and (b) poly(BPVS-Pan-*ran*-NVC) in $CDCl_3$.

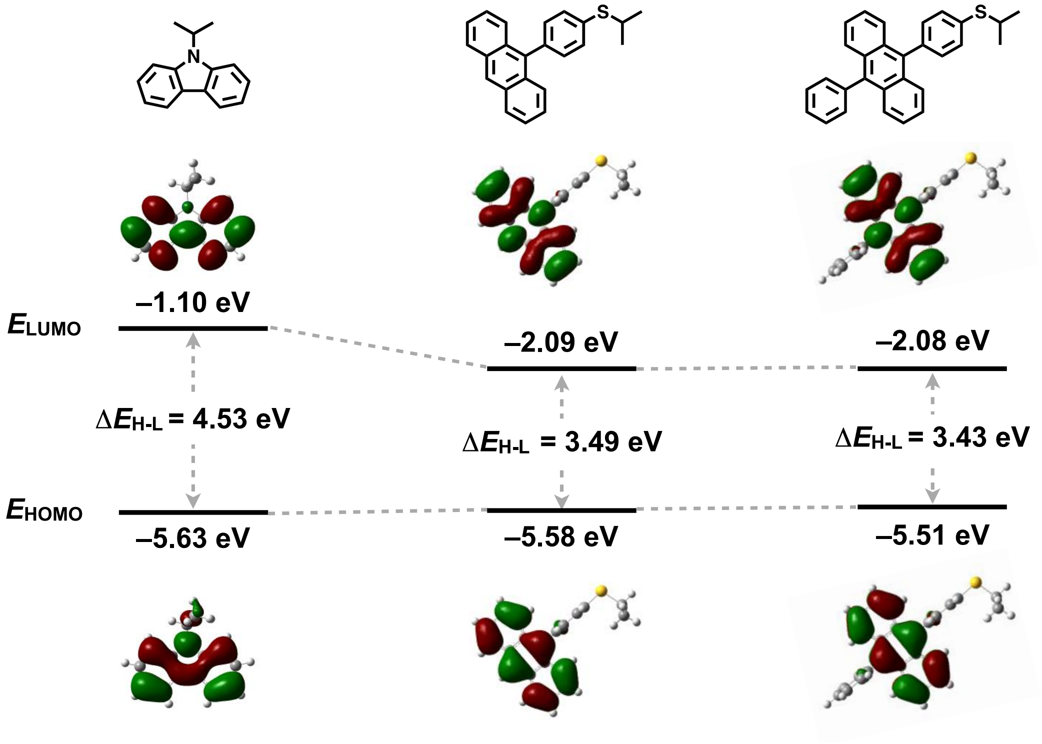

Figure S4. Spatial distributions of the HOMOs and LUMOs of carbazole and (di)phenylanthracene moieties.

Table S1. Synthesis of poly(BPVS)-*b*-poly(NVC) at 60 ºC for 24 h in bulk [a)].

Entry	Macro-CTA	$[M]_0$ [a)] /[Macro-CTA]$_0$	Yield [b)] (%)	M_n [c)] (theory)	M_n [d)] (EA)	M_n [e)] (SEC)	M_w/M_n [e)] (SEC)	Composition [e)] BPVS : NVC
1	poly(BPVS)	100	81	27,900	28,100	19,400	1.28	41 : 59

[a)] [Macro-CTA]$_0$/[AIBN]$_0$ = 2/1. [b)] Hexane-insoluble part. [c)] The theoretical molecular weight ($M_{n,\text{ theory}}$) = (M_w of NVC) × [NVC]$_0$/[Macro-CTA]$_0$ × yield + (M_w of Macro-CTA).[d)] Calculated by elemental analysis (EA). [e)] Measured by size-exclusion SEC using polystyrene standards in DMF (10 mM LiBr). [f)] P(BPVS) macro-CTA : $M_{n,\text{ NMR}}$ = 12,200, $M_{n,\text{ SEC}}$ = 7,700, M_w/M_n = 1.36.

Table S2. RAFT copolymerization of BPVS with NVC using xanthate-type CTA[a].

Entry	$[BPVS]_0$/ $[NVC]_0$	Yield [b] (%)	M_n [c] (SEC)	M_w/M_n [c] (SEC)	Composition [d] M1 : M2
1	50/150	52	8,200	1.40	40 : 60

[a] $[CTA]_0/[AIBN]_0 = 2/1$, $[M1+M2]_0 = 10M$. [b] Hexane-insoluble part. [c] Number-average molecular weight (M_n) and molecular weight distribution (M_w/M_n) were measured by size-exclusion chromatography (SEC) using polystyrene standards in DMF (10 mM LiBr). [d] Calculated by elemental analysis.

Table S3. Solubility of poly(BPVS-An), poly(BPVS-PAn), and poly(NVC).

Sample	H_2O	Acetone	THF	MeOH	EtOH
poly(BPVS-An)	-	-	+	-	-
poly(BPVS-PAn)	-	-	+	-	-
poly(NVC)	-	-	+	-	-

Sample	Ether	Chloroform	EtOAc	DMF	Hexane
poly(BPVS-An)	-	+	-	+	-
poly(BPVS-PAn)	-	+	-	+	-
poly(NVC)	-	+	-	+	-

+ : Soluble at room temperature, - : Insoluble at room temperature.
